# K^+ ^channel openers restore verapamil-inhibited lung fluid resolution and transepithelial ion transport

**DOI:** 10.1186/1465-9921-11-65

**Published:** 2010-05-27

**Authors:** Dong-Yun Han, Hong-Guang Nie, Xiu Gu, Ramesh C Nayak, Xue-Feng Su, Jian Fu, Yongchang Chang, Vijay Rao, Hong-Long Ji

**Affiliations:** 1 Department of Biochemistry, University of Texas Health Science Center at Tyler, Tyler, TX 75708, USA; 2 Texas Lung Injury Institute, University of Texas Health Science Center at Tyler, Tyler, TX 75708, USA; 3 Division of Neurobiology, Barrow Neurological Institute, St Joseph's Hospital and Medical Center, Phoenix, AZ 85013, USA; 4 Department of Pharmacology, School of Pharmaceutical Sciences, China Medical University, Shenyang 110001, Liaoning, China; 5 Division of Respiratory Diseases, Department of Internal Medicine, Second Teaching Hospital, China Medical University, Shenyang 110001, Liaoning, China

## Abstract

**Background:**

Lung epithelial Na^+ ^channels (ENaC) are regulated by cell Ca^2+ ^signal, which may contribute to calcium antagonist-induced noncardiogenic lung edema. Although K^+ ^channel modulators regulate ENaC activity in normal lungs, the therapeutical relevance and the underlying mechanisms have not been completely explored. We hypothesized that K^+ ^channel openers may restore calcium channel blocker-inhibited alveolar fluid clearance (AFC) by up-regulating both apical and basolateral ion transport.

**Methods:**

Verapamil-induced depression of heterologously expressed human αβγ ENaC in *Xenopus *oocytes, apical and basolateral ion transport in monolayers of human lung epithelial cells (H441), and *in vivo *alveolar fluid clearance were measured, respectively, using the two-electrode voltage clamp, Ussing chamber, and BSA protein assays. Ca^2+ ^signal in H441 cells was analyzed using Fluo 4AM.

**Results:**

The rate of *in vivo *AFC was reduced significantly (40.6 ± 6.3% of control, *P *< 0.05, n = 12) in mice intratracheally administrated verapamil. K_Ca3.1 _(1-EBIO) and K_ATP _(minoxidil) channel openers significantly recovered AFC. In addition to short-circuit current (Isc) in intact H441 monolayers, both apical and basolateral Isc levels were reduced by verapamil in permeabilized monolayers. Moreover, verapamil significantly altered Ca^2+ ^signal evoked by ionomycin in H441 cells. Depletion of cytosolic Ca^2+ ^in αβγ ENaC-expressing oocytes completely abolished verapamil-induced inhibition. Intriguingly, K_V _(pyrithione-Na), K _Ca3.1 _(1-EBIO), and K_ATP _(minoxidil) channel openers almost completely restored the verapamil-induced decrease in Isc levels by diversely up-regulating apical and basolateral Na^+ ^and K^+ ^transport pathways.

**Conclusions:**

Our observations demonstrate that K^+ ^channel openers are capable of rescuing reduced vectorial Na^+ ^transport across lung epithelial cells with impaired Ca^2+ ^signal.

## Background

Drug-induced noncardiogenic lung edema is one of the pulmonary manifestations of the life-threatening side effects resulting from an overdose of medicines. All four subgroups of calcium channel blockers (CCB) have been reported to lead to both cardiogenic and noncardiogenic pulmonary edema [1-8]. CCB-induced noncardiogenic edema appears to be due to diffuse damage and increased permeability of the alveolocapillary membrane, which results in accumulation of excess fluid in alveolar air spaces [9]. To keep the alveolar space free from flooding, accumulated cytosolic salts are extruded [10-12]. The major determinant pathway for this process is apically located epithelial Na^+ ^channels (ENaC). Increasing amounts of etiological evidence suggests that genetic and pathologic ENaC deficiency gives rise to the genesis of flooding airspaces [13,14]. For example, α ENaC knockout leads to the death of newborn mice due to their inability to resolve amniotic fluid in their lungs [15]. In adult lungs, high attitude pulmonary edema and pathogen-challenged edematous lung injuries have been linked to a reduction of both ENaC expression and activity levels [16,17].

Basolateral K^+ ^channels in epithelia play a major role in maintaining the electrochemical gradient necessary for Na^+ ^and Cl^- ^transepithelial transport, and in restoring the resting membrane potential. The potential physiological importance of voltage-gated K^+ ^channels (K_V_), calcium-activated K^+ ^channels (K_Ca_), and ATP-sensitive K^+ ^channels (K_ATP_) in transepithelial ion transport has been implicated [18-22]. K_V _channels constitute a large family (*i.e*., K_V_LQT1-K_V_7.1, KNCQ, and KCNQ channels). So far, KCNQ 3 and 5 but not 1 have been identified in H441 cells by a very recent publication [23]. K_Ca _channels, until recently known as K _Ca3.1 _and BK_Ca_, are functionally detected in ENaC-expressing primary airway and ATII cells [24-26]. These commonly basolaterally located K _Ca3.1 _channels are blocked by clotrimazole and are activated by 1-ethyl-2-benzimidazolinone (1-EBIO). K_ATP _channels, which can be inhibited by glibenclamide and activated by minoxidil, have been identified in both fetal and adult alveolar cells [21,27]. These three types of K^+ ^channels have been confirmed to functionally modify the ionic and fluid transepithelial transport in cystic fibrosis airway epithelial cells [22] and may have an important role in lung fluid clearance [21,28]. These crucial K^+ ^channels together with basolaterally located Na^+^/K^+^-ATPase recycle K^+ ^ions across interstitial membrane of alveolar cells. The regulation of transepithelial Na^+ ^transport by the K^+ ^channel blockers in normal primary alveolar type II cells has recently been reported [21,25]. The underlying mechanisms for the coupling of Na^+ ^and K^+ ^transport are unknown. More importantly, K^+ ^channel openers facilitated alveolar fluid clearance in resected human lungs [29] and transepithelial ion transport in human airway [30]. However, whether K^+ ^channel openers are able to restore the CCB-inhibited transepithelial salt and fluid clearance in edematous lungs remains to be elucidated.

Verapamil has been broadly used clinically for combating hypertension, ischemic heart diseases, supraventricular tachyarrhythmias, and tycolysis. In this study, we investigated the effects of verapamil on ENaC activity in confluent H441 monolayers-a human bronchoalveolar epithelial cell line, in *Xenopus *oocytes heterologously expressing human αβγ ENaC, and in murine lungs. Our results showed that K^+ ^channel openers recovered verapamil-inhibited vectorial Na^+ ^transport in H441 cells. Moreover, verapamil-reduced alveolar fluid resolution can be restored by these K^+ ^channel openers in murine lungs.

## Methods

### Cell culture

NCI-H441 (H441) cells were obtained from the American Type Culture Collection (ATCC). H441 cells were grown in RPMI medium (ATCC) containing 10% fetal bovine serum (FBS), 2 mM L-glutamine, 10 mM HEPES, 1 mM sodium pyruvate, 4.5 g/L glucose, 1.5 g/L sodium bicarbonate and antibiotics (100 U/ml penicillin and 100 μg/ml streptomycin). Dexamethasone (250 nM, Sigma) was supplemented to stimulate ENaC expression. Cells were seeded on permeable support filters (Costar) at a supraconfluent density (~5 × 10^6 ^cells/cm^2^), and incubated in a humidified atmosphere of 5% CO_2_-95% O_2 _at 37°C. Cells reached confluency in the Costar Snapwell culture cups 24 hrs after plating. At this point media and non-adherent cells in the apical compartment were removed to adapt the cells to air-liquid interface culture. Culture media in the basolateral compartment was replaced every other day; whereas the apical surface was rinsed with PBS. An epithelial tissue voltohmmeter (World Precision Instruments) was used to monitor the transepithelial resistance. Highly polarized tight monolayers with resistance >800 Ω·cm^2 ^were selected for Ussing chamber assays.

### *In vivo *alveolar fluid clearance

Animals were kept under pathogen-free conditions, and all procedures performed were approved by the Institutional Animal Care and Use Committee of the University of Texas Health Science Center at Tyler. Alveolar fluid clearance was examined *in vivo *as previously described by us and other groups [31-34]. Briefly, 8-10 week old, weighting 20-30 g, pathogen-free, male C57/BL/6 mice were used (National Cancer Institute). An isosmotic instillate containing 5% bovine serum albumin (BSA) was prepared with 0.9% NaCl. Anesthetized mice were ventilated with 100% O_2 _via a volume-controlled ventilator (model 683, Harvard Apparatus) for a 30-minute period. 5% BSA (0.3 ml), with or without verapamil (100 μM) and amiloride (1 mM) was instilled intratracheally. The instilled alveolar fluid was aspirated by applying gentle suction to the tracheal catheter with a 1-ml syringe. The BSA content of the alveolar fluid was measured with a 96-well microplate reader. Alveolar fluid clearance (AFC) was calculated as follows: AFC = (Vi - Vf)/Vi*100, where Vi and Vf denote the volume of the instilled and recovered alveolar fluid, respectively. Vf was obtained as Vf = (Vi * Pi )/Pf, where Pi and Pf represent protein concentration of instilled and collected fluid.

### Ussing chamber assays

Measurements of short-circuit current (Isc) in H441 monolayers were performed as described previously [35]. Briefly, H441 monolayers were mounted in vertical Ussing chambers (Physiologic Instruments) and bathed on both sides with solutions containing (in mM) 120 NaCl, 25 NaHCO_3_, 3.3 KH_2_PO_4_, 0.83 K_2_HPO_4_, 1.2 CaCl_2_, 1.2 MgCl_2_, 10 HEPES, 10 mannitol (apical compartment) and 10 glucose (basolateral compartment). Each solution was iso-osmolalic (approximately 300 mmol/Kg), as measured by a freezing depression osmometer (Wescor). The transepithelial Isc levels were measured with 3 M KCl, 4% agar bridges placed 3 mm on either side of the membrane, which were connected on either side to Ag-AgCl electrodes. The filters were bathed on both sides with the above salt solution as designed, bubbled continuously with a 95% O_2_-5% CO_2 _gas mixture (pH 7.4). The temperature of the bath solution (37°C) was maintained using a water bath. The transmonolayer potential was short-circuited to 0 mV, and Isc level was measured with an epithelial voltage clamp (VCC-MC8, Physiologic Instruments). A 10-mV pulse of 1s duration was imposed every 10s to monitor Rt. Data were collected using the Acquire and Analyse program (version 2.3; Physiologic Instruments). When Isc level reached plateau, drugs were pipetted to the either apical or basolateral compartment.

To determine whether verapamil decreases the amiloride-sensitive Isc level across the apical membrane, 100 μM amphotericin B, a pore-forming antibiotic (Sigma), was added to the basolateral side of Ussing chamber to permeabilize the basolateral membrane [36]. A 145:25 mM Na^+ ^ionic gradient (apical to basolateral compartment) was established by replacing 120 mM Na^+ ^ions with equal molar N-methyl-D-glucamine, an impermeant cation in the basolateral bath solution. Basolateral permeabilization equilibrates intracellular Na^+ ^concentration to 25 mM in the basolateral bath. To exclude any potentially residual Na^+^/K^+^-ATPase activity, 1 mM ouabain was added to the interstitial compartment. Under these experimental conditions, amiloride-sensitive Isc level reflects passive electrogenic Na^+ ^movement through ENaC down the Na^+ ^concentration gradient [37,38]. When Isc level had attained its stable level, verapamil was applied to the apical side and amiloride-sensitive current component was determined by adding 100 μM amiloride.

To examine the ouabain-inhibitable Isc level across the basolateral membrane, the apical membrane was permeabilized with 10 μM amphotericin B. Apical permeabilization loads the cytosol with Na^+ ^ions thereby eliciting the maximal active Na^+ ^transport by the Na^+^/K^+^-ATPase [39]. To eliminate any remaining ENaC activity, 100 μM amiloride was included in the apical bath. Under these experimental conditions, ouabain-inhibitable basolateral Isc shall associate with Na^+^/K^+^-ATPase, tightly coupling with K^+ ^channels. When the Isc level was stable, verapamil and K^+ ^channel modulators were applied. To determine Na^+^/K^+^-ATPase activity, 1 mM ouabain was added to the basolateral compartment at the end of recording.

### Oocyte preparation and voltage clamp analysis

Oocytes were surgically removed from appropriately anesthetized adult female *Xenopus laevis *(Xenopus Express) and cRNAs for human α, β, and γ ENaC were prepared as described previously [40]. Briefly, the ovarian tissue was removed from frogs under anesthesia by ethyl 3-aminobenzoate methanesulfonate salt (Sigma) through a small incision in the lower abdomen. Follicle cells were removed and digested in OR-2 Ca^2+^-free medium (in mM: 82.5 NaCl, 2.5 KCl, 1.0 MgCl_2_, 1.0 Na_2_HPO_4_, and 10.0 HEPES, pH 7.5) with the addition of 2 mg/ml collagenase (Roche Indianapolis). Defolliculated oocytes were cytosolically injected with ENaC cRNAs (25 ng) per oocyte in 50 nl of RNase free water and incubated in half-strength L-15 medium at 18°C for 48 h. Oocytes were impaled with two electrodes filled with 3 M KCl, having resistances of 0.5-2 MΩ. A TEV-200 voltage clamp amplifier (Dagan) was used to clamp oocytes with concomitant recording of currents. The continuously perfused bathing solution was ND96 medium (in mM: 96.0 NaCl, 1.0 MgCl_2_, 1.8 CaCl_2_, 2.5 KCl, and 5.0 HEPES, pH 7.5). To prepare a Ca^2+^-free bath solution, CaCl_2 _was omitted and 5 mM EGTA was added. To chelate intracellular Ca^2+ ^ions, 10 μM BAPTA_AM was added to the Ca^2+^-free bath solution. Experiments were controlled by pCLAMP 10.1 software (Molecular Devices), and currents at -40, -100, and +80 mV were continuously monitored with an interval of 10 s. Data were sampled at the rate of 1,000 Hz and filtered at 500 Hz.

### Fluo 4 AM measurements

Intracellular Ca^2+ ^signal elicited by ionomycin in epithelial cells was measured as described previously [41-44]. H441 cells were grown on chambered coverglass for 48 h. Culture medium was aspirated and cells were loaded with cell permeable Fluo 4 AM dye (4 μM, Invitrogen, CA) for 1 h. The Fluo 4 AM loaded cells were then incubated with verapamil or culture medium for 10 min. The cells were placed on the specimen stage of an inverted microscope (AxioObserver Z1, Carl Zeiss) equipped with a LSM 510 Meta confocal system (Carl Zeiss, Germany). The argon ion 488 nm laser line was used to excite Fluo 4 AM fluorochrome and the serial live cell images for the emission signal of Fluo 4 AM were captured for a period of 6 min 40 s at an interval of 4 s using a 20 ×/0.8 Plan-apochromate objective lens. Subsequent to a 2 min image acquisition, 15 nM ionomycin was added into the chamber to evoke an increment in cytosolic Ca^2+ ^signal. In all cases, a confluent field of cells was chosen for imaging. The relative Ca^2+ ^signal was measured as the ratio of fluorescent intensity (F/F0) using ZEN 2007 Zeiss imaging software and plotted as a function of recording time.

### Statistics

Electrophysiological data from Ussing chamber and voltage-clamp studies were primarily analyzed with the Acquire and Analyze 2.3 (Physiologic Instruments) and Clampfit 10.1 (Molecular Devices), respectively. The measurements were then imported into OriginPro 8.0 (OriginLab) for statistical computation and graphic plot. The IC_50 _and EC_50 _values of verapamil and K^+ ^channel openers were calculated by fitting the dose-response curves with the Hill equation.

All results are presented as mean ± S.E.M. The unsorted data were examined for the normal distribution using either the Kolmogorov-Smirnov normality test with specified parameters previously published or Lilliefors test. Those without significantly drawn from the normally distributed population were selected for t-test and ANOVA analyses. For the comparison of mean values of repeated measures of short-circuit and whole-cell activities, paired two-tailed Student t-test was used. For unpaired electrophysiological data, one-way ANOVA analysis combined with a post hoc Tukey-Kramer test was used. For analyses of *in vivo *alveolar fluid clearance, mean values between control and CCB challenged groups were compared by the unpaired two-sample Student t-test for both equal variance assumed or not. The mean and SE values of amiloride-sensitive AFC fraction were computed using the following equations:

and

where *M_t _*and *M_a _*are mean values of total and amiloride-resistant fractions; *t_c _*is the t_.95 _value of a freedom of (*n_t_+n_a_-2*) in the t-table; *SE_t _*and *SE_a _*are SE values of total and amiloride-resistant AFC. *M, SE*, and *n *stand for mean, standard error, and number of mice, respectively. For nonparametric data (*i.e*., Ca^2+ ^signal), the Mann-Whitney U-test was used. The power of sample size was simultaneously evaluated to assure the actual power value > 0.95. *P *< 0.05 was considered statistically significant.

## Results

### Verapamil reduces murine *in vivo *fluid resolution

**Figure 1:**
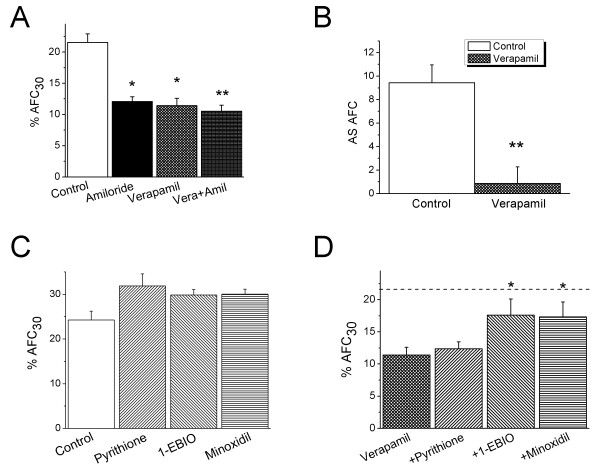
**Recovery of verapamil-reduced alveolar fluid clearance (AFC) by K^+ ^channel openers *in vivo***. **(A) **Verapamil intratracheal application reduces alveolar fluid clearance. Verapamil (100 μM) was intratracheally delivered to mouse lung. Average AFC values in the absence of drugs (Control), in the presence of amiloride (Amiloride), verapamil (Verapamil), and both (Amiloride+Verapamil). Unpaired two-sample two-tailed Student t-test. *P < 0.05 and **P < 0.01 when compared with Control. n = 4-15. **(B) **Amiloride-sensitive (AS) AFC. The mean and SE values were computed as described in Methods. Unpaired two-sample two-tailed Student t-test. **P < 0.01. n = 12-15. **(C) **Effects of K^+ ^channel openers on basal AFC. Unpaired two-sample two-tailed Student t-test. n = 5-15. **(D) **K^+ ^channel openers restore verapamil-reduced AFC. AFC values were measured for Verapamil (100 μM) alone, + Pyrithione-Na (1 mM), + 1-EBIO (1 mM), and +Minoxidil (0.6 mM). The dashed line indicates the Control level. Unpaired two-sample two-tailed Student t-test. *P < 0.05 *vs *Verapamil alone. n = 4-12.

To examine the potential deleterious effects of calcium channel blockers (CCB) on fluid resolution in distal lung air spaces, we measured *in vivo *alveolar fluid clearance (AFC) in anesthetized C57/B6 mice. As plasma verapamil predominately affects cardiovascular function, which may lead to both cardiogenic and noncardiogenic pulmonary edema as reported clinically [1-8], we intratracheally delivered verapamil into lung to avoid any dysfunction beyond air spaces. As shown in Fig. 1A, the normal AFC rate was 23.6 ± 1.3% (n = 15). Intratracheal instillation of verapamil (100 μM) markedly reduced the re-absorption of the 5% BSA instillate (11.4 ± 1.2%, *P *< 0.05, n = 12), which was almost identical to that in the presence of amiloride (1 mM, 12.1 ± 0.8%, n = 4, *P *< 0.05 *vs *Control). In the presence of both amiloride and verapamil, fluid resolution was 10.6 ± 0.9% (*P *< 0.01 *vs *Control, n = 4), suggesting that verapamil almost completely inhibited amiloride-sensitive fraction of AFC (Fig. 1B). These *in vivo *data clearly demonstrate that CCB impairs transalveolar fluid clearance, which in turn results in fluid accumulation in lung sacs.

### K^+ ^channel openers profoundly restore verapamil-inhibited alveolar fluid clearance

K^+ ^channel openers activated transepithelial ion transport in alveolar monolayers *in vitro *under physiological conditions [25]. It prompted us to hypothesize that K^+ ^channel openers may be capable of recovering the verapamil-inhibited fluid resolution *in vivo*. To address this promising pharmaceutical issue, three types of K^+ ^channel openers, namely, pyrithione-Na (1 mM for K_V_), 1-EBIO (1 mM for K _Ca3.1_), and minoxidil (0.6 mM for K_ATP_) were intratracheally delivered in the presence (Fig. 1D) and absence of verapamil (Fig. 1C). The K^+ ^openers slightly but not significantly altered AFC (Fig. 1C). In sharp contrast, depressed AFC (10.4 ± 1.3%) in the presence of verapamil was pronouncedly relieved by 1-EBIO (17.6 ± 2.5%, n = 4, *P *< 0.05) and minoxidil (17.3 ± 2.3%, n = 4, *P *< 0.05). These data suggest that augmentation of K^+ ^efflux from lung epithelial cytosol facilitates salt/fluid re-absorption in verapamil-injured edematous lungs.

### Calcium antagonists abrogate transepithelial short-circuit current (Isc) in intact H441 monolayers

**Figure 2:**
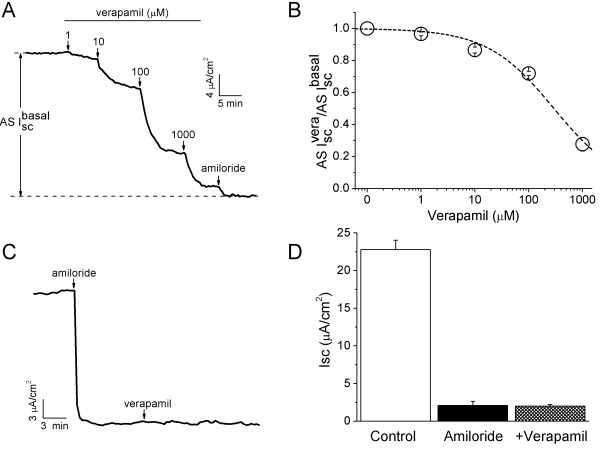
**Verapamil reduces short-circuit (Isc) level in H441 monolayers in a dose-dependent manner**. **(A) **Representative Isc trace showing applications of a series of concentrations. Amiloride-sensitive Isc level (AS I^basal^_sc_) is the sum of verapamil-inhibitable and residual amiloride-sensitive fractions. **(B) **Normalized AS Isc levels (AS I^vera^_sc_/AS I^basal^_sc_) at each concentration were plotted as a dose-response curve. n = 6. The raw data were fitted with the Hill equation. IC_50 _value, 294.2 μM. **(C) & (D) **Verapamil on amiloride-insensitive Isc levels in amiloride pretreated cells. Representative trace **(C) **and corresponding average Isc levels before (Control) and after addition of amiloride and verapamil **(D)**. n = 3. *P *= 0.89 for the Isc levels before and after verapamil. Paired t-test.

Human bronchoalveolar epithelium-derived Clara cells (H441) have been used extensively to study lung epithelial Na^+ ^channels, in which ENaC properties are similar to those in primary alveolar type II cells [45-48]. To examine the effects of verapamil on the electrogenic transepithelial Na^+ ^transport in lung epithelial cells, confluent H441 monolayers were mounted in an 8-chamber Ussing chamber system. Verapamil inhibited Isc levels when applied to the luminal side of H441 monolayers in a dose-dependent manner (Fig. 2A). The IC_50 _value was 294.2 μM calculated by fitting the dose-response curve with the Hill equation (Fig. 2B). Nevertheless, verapamil did not affect the Isc levels in amiloride-exposed monolayers (Fig. 2C &2D, before 2.1 ± 0.6 μA/cm^2 ^and after verapamil 2.0 ± 0.2 μA/cm^2^, *P*>0.05, n = 3). These results suggest that verapamil inhibits vectorial transepithelial ion transport in a dose-dependent manner in intact monolayers.

**Figure 3:**
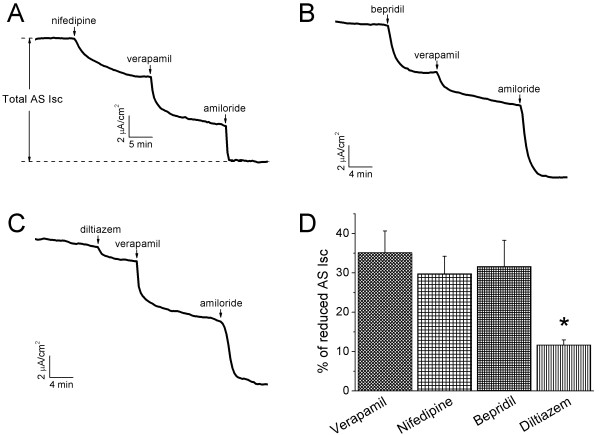
**Effects of CCB compounds on transepithelial short-circuit currents (Isc) in intact H441 monolayers**. **(A-C) **Typical traces. 200 μM nifedipine **(A)**, 10 μM bepridil **(B) **or 50 μM diltiazem **(C) **was added to the basolateral compartment followed by verapamil. Amiloride (100 μM, apical side) was finally applied to inhibit residual amiloride-sensitive currents. Arrows show the time point of addition. Total AS Isc is the difference between the Isc level before CCB and the amiloride-insensitive fraction, as indicated by a pair of vertical arrows. **(D) **CCB-sensitive fraction: CCB-inhibitable Isc/total AS Isc. One-way ANOVA. *P < 0.05 *vs *Verapamil. n = 3-10.

To measure the regulation of ENaC-associated transepithelial Isc levels by representative examples from the other three subgroups of CCB compounds, confluent H441 monolayers were exposed to nifedipine, bepridil, and diltiazem (Fig. 3). As shown by the representative current traces, a reduction in the Isc levels was recorded following bolus addition of nifedipine (200 μM), bepridil (10 μM), or diltiazem (50 μM) (Fig. 3A-C). To compare the inhibitory efficacy of these four subgroups of CCB compounds, verapamil (100 μM) was applied subsequently to these CCB compounds. Interestingly, verapamil resulted in a further decrease in the Isc levels. On average, nifedipine, bepridil, and diltiazem inhibited amiloride-sensitive (AS) Isc levels by 29.8 ± 4.4% (*P *< 0.01, n = 4), 31.6 ± 6.6% (*P *< 0.01, n = 3), and 11.7 ± 1.3% (*P *< 0.01, n = 3), respectively (Fig. 3D). Subsequent addition of verapamil to each group showed a further reduction in the Isc levels to approximately the same level of 70% of total reduction (Fig. 3). Because verapamil displayed potent inhibition on the AS Isc levels in H441 cells, this drug was then used for the follow-up experiments.

**Figure 4:**
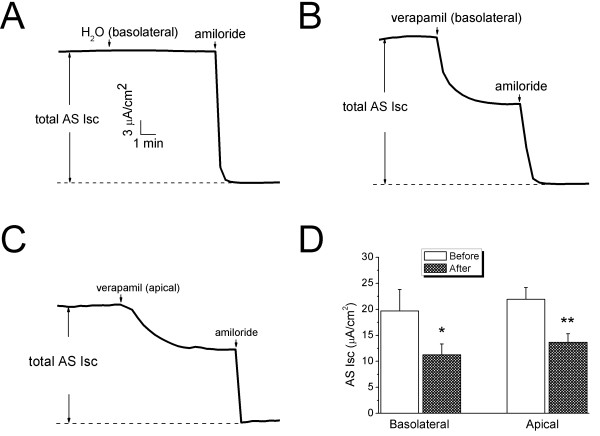
**Comparison of verapamil-inhibitable Isc levels in H441 monolayers when applied to the apical or basolateral compartments**. **(A-C) **Representative Isc traces showing the effects of water (H_2_O), verapamil applied to basolateral **(B) **and apical **(C) **compartments. The total AS Isc levels associated with ENaC are designated by pairs of vertical arrows. **(D) **AS Isc levels before and after verapamil delivery to the basolateral side or apical compartment. Paired t-test for comparison of current levels before and after verapamil. *P < 0.05 and **P < 0.01. n = 4-17.

Verapamil, as well as other CCB compounds, is cell permeable and therefore may cross the thin alveolocapillary membrane and exhibit its inhibitory effects in the alveolar space. To investigate whether or not verapamil has the same effects on the Isc levels when applied to the basolateral and apical sides, we performed a set of experiments by adding verapamil (100 μM) to either basolateral or apical compartment (Fig. 4). AS Isc levels were inhibited by both basolateral and apical addition of verapamil by 41.4 ± 2.6% and 38.8 ± 1.7%, respectively (Fig. 4D, n = 4-17). However, addition of the same volume of water did not alter Isc level (Fig. 4A). These data suggest that verapamil reduces AS Na^+ ^channels to a similar extent regardless of its application to either luminal or interstitial compartment.

### Verapamil inhibits both apical and basolateral Na^+ ^conductance in permeabilized H441 monolayers

**Figure 5:**
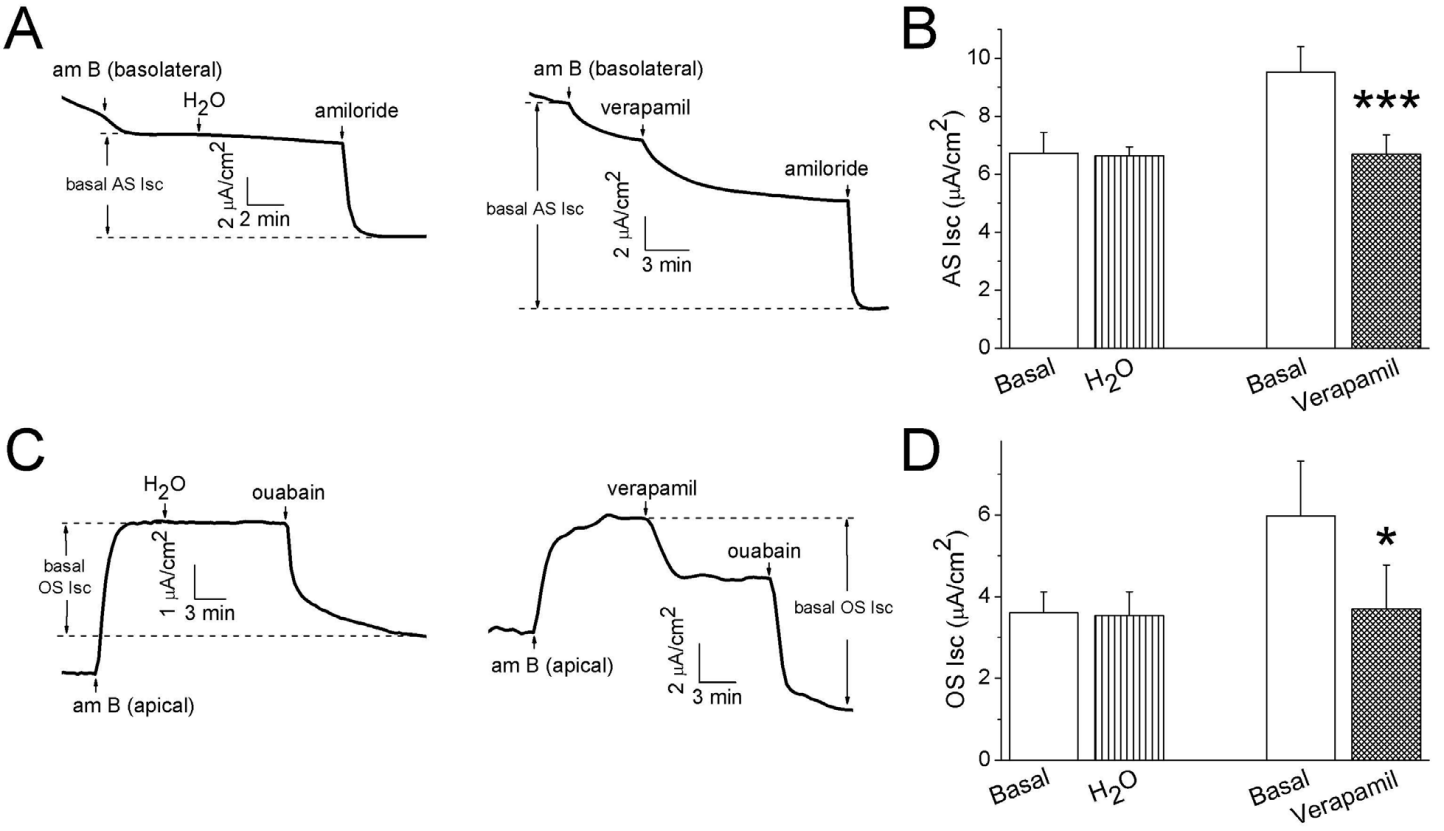
**Inhibition of transapical and transbasolateral Isc levels by verapamil in permeabilized H441 monolayers**. **(A) **Representative Isc traces obtained in basolateral permeabilized H441 monolayers with amphotericin B (am B). 1 mM ouabain was added to the basolateral side to exclude any potential residual Isc across basolateral membrane. Amiloride (100 μM) was applied at the end of recording to calculate basal amiloride-sensitive (AS) Isc level as indicated between dashed lines. **(B) **AS Isc levels before (Basal) and after water (H_2_O) and verapamil (Verapamil). Paired t-test. ***P < 0.001. n = 4-8. **(C) **Representative Isc trace recorded in apically permeabilized H441 monolayers with amphotericin B. 100 μM amiloride was added to the apical compartment to inhibit possible residual Isc level carried by ENaC. Ouabain (1 mM) was added at the end of the recording to calculate total ouabain-sensitive (OS) Isc level. **(D) **Mean OS Isc levels in the absence (Basal) and presence of water (H_2_O) and verapamil (Verapamil). Paired t-test. *P < 0.05. n = 4.

It has been reported that the total Na^+ ^Isc level in polarized lung epithelial monolayers is predominately determined by apical and basolateral vectorial Na^+ ^movement [13]. We asked whether verapamil might regulate electrogenic pathways across both apical and basolateral membrane. To examine the effects of verapamil on apical Na^+ ^influx, amphotericin B (100 μM) was applied to permeabilize the basolateral membrane (Fig. 5A). A large Na^+ ^ion gradient was applied to the permeabilized H441 monolayer to facilitate passive Na^+ ^transport predominately through ENaC channels. To confidentially eliminate all of Na^+^/K^+^-ATPase enzymatic activity, ouabain (1 mM) was added to the basolateral compartment. Permeabilization of the basolateral membrane caused a reduction in the Isc level, suggesting that a relatively larger Na^+ ^gradient across apical membrane exists in intact cells (apical 145:~10 mM in cytosol) than basolateral permeabilized monoalyers (145:25 mM). Verapamil inhibited transapical AS Isc levels from 9.5 ± 0.9 to 6.7 ± 0.8 μA/cm^2 ^(paired t-test, *P *< 0.001, n = 8, Fig. 5B). Clearly, verapamil regulates AS apical Na^+ ^conductance in the absence of cytosolic soluble signal elements.

We then examined the effects of verapamil on Na^+^/K^+^-ATPase in apically permeabilized confluent H441 monolayers with amphotericin B (10 μM). To eliminate possibility of any AS apical Na^+ ^channels still remaining in the apically permeabilized cells, amiloride (100 μM) was added to the apical compartment. As shown in Fig. 5C, in the presence of amiloride, apical permeabilization caused a dramatic increase in the Isc level, a hallmark of evoked Na^+^/K^+^-ATPase activity following an increment in "cytosolic" Na^+ ^ions. Verapamil resulted in a marked drop of the ouabain-sensitive (OS) Isc level from 6.0 ± 1.3 to 3.7 ± 1.1 μA/cm^2 ^(*P *< 0.05, n = 4, Fig. 5D). These experiments provide direct evidence that verapamil inhibits Na^+^/K^+^-ATPase in the apically permeabilized H441 cells.

### Verapamil serves as a K^+ ^channel blocker

**Figure 6:**
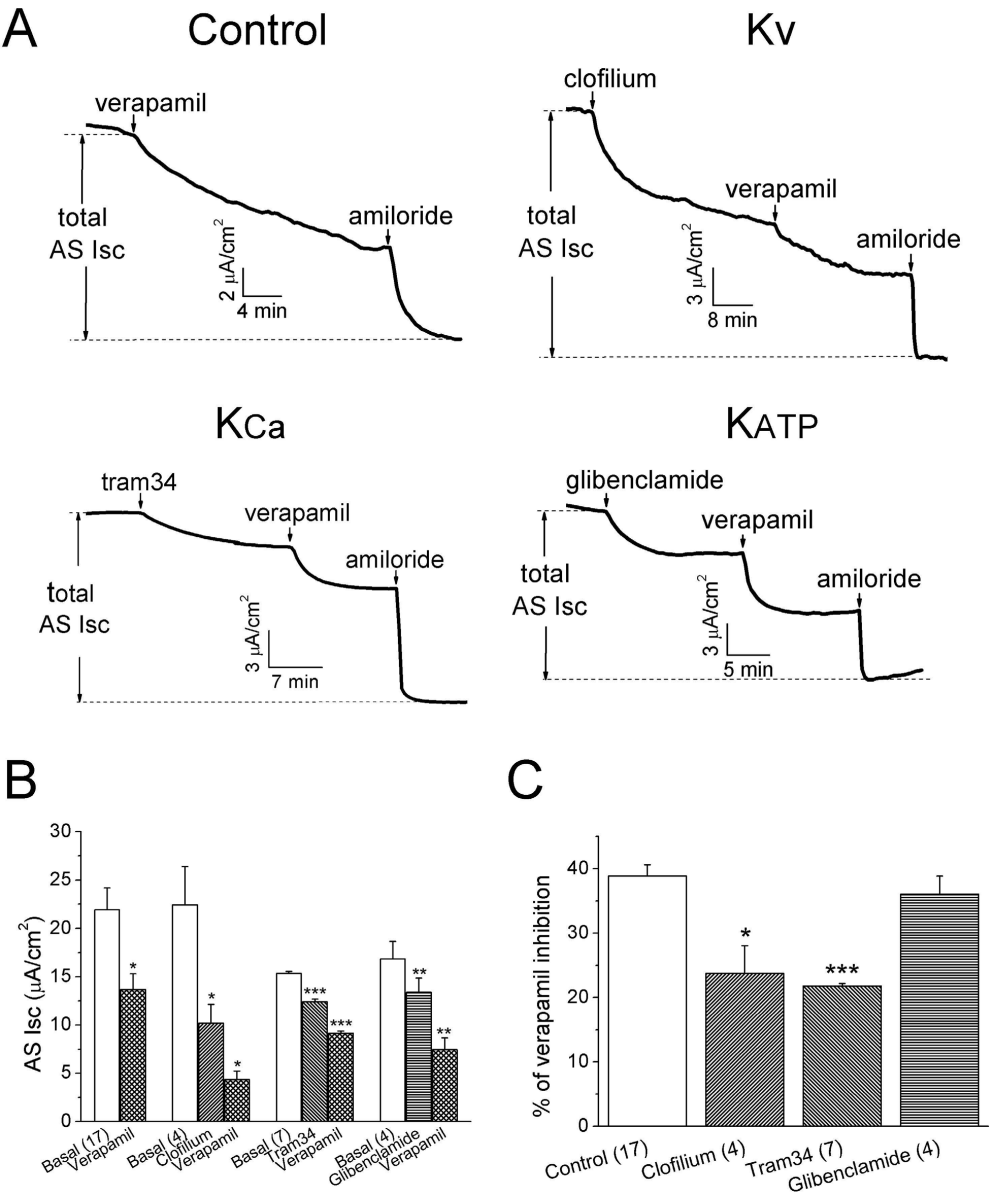
**K^+ ^channel blockers alter the inhibitory effects of verapamil in H441 cells**. **(A) **Typical Isc traces showing the application of 100 μM verapamil alone (control), 100 μM clofilium (K_V _inhibitor), 20 μM tram34 (K_Ca3.1 _inhibitor), and 100 μM glibenclamide (K_ATP _inhibitor), respectively. These K^+ ^channel blockers were applied to basolateral side followed by verapamil and amiloride (apical side) to compute total AS Isc. **(B) **Summary of average AS Isc levels. Paired t-test. *P < 0.05, **P < 0.01, *** *P *< 0.001 for comparison of pre- and post exposure of CCB. n = 4-17. **(C) **Reduced percentages of AS Isc levels by verapamil in H441 cells with and without pretreatment of K^+ ^channel blockers. Two-sample, two-tailed t-test. *P < 0.05 *vs *Control. n = 4-17.

Verapamil has been known to alter cytosolic Ca^2+ ^concentration and to modify a number of K^+ ^channels [49]. We hence speculated that verapamil might indirectly influence ENaC activity by altering K^+ ^channels. The basolateral K^+ ^channels tightly regulate Na^+^/K^+^-ATPase activity, by coordinately acting as the K^+ ^recycling machinery to maintain the negative resting membrane potential. Resultant depolarization of polarized epithelial cells, a consequence of impaired K^+ ^recycling, weakens the electrochemical driving force for ENaC activity. We thereby attempted to determine the individual contribution of each functional subtype of K^+ ^channels (K_V_, K_Ca3.1 _and K_ATP_) to verapamil-inhibited ENaC activity. The representative Isc traces showed the verapamil-induced decrease in AS Isc subsequent to addition of 100 μM clofilium, 5 μM tram34, and 100 μM glibenclamide, respectively (Fig. 6A). These concentrations were supposed to completely block corresponding K^+ ^channels as described previously [21,25]. As summarized in Fig. 6B, clofilium, tram34, and glibenclamide decreased the AS Isc levels by 54.4 ± 4.6% (*P *< 0.05, n = 4), 19.1 ± 1.8% (*P *< 0.001, n = 7), 20.5 ± 1.1% (*P *< 0.01, n = 4), respectively. Subsequent addition of verapamil resulted in a further reduction of the residual AS Isc levels by 23.7 ± 4.3%, 40.3 ± 1.6%, and 36.0 ± 2.8%, respectively. Blockade of KCNQ (3 and 5) [23] but not K_Ca3.1 _and K_ATP _channels significantly affected the response of AS Na^+ ^channels to verapamil (Fig. 6C, *P *< 0.05), when compared to the control (38.8 ± 1.7%, n = 17). Our results showed that these three subtypes of K^+ ^channels are functionally expressed in H441 cells at a various levels, in accordance with other studies [21,25]. Moreover, inhibition of these K^+ ^channels by the related specific blockers can influence the inhibitory effects of verapamil on AS Na^+ ^channels to various extents.

### K^+ ^channel openers restore verapamil-inhibited Isc levels in intact H441 cells

**Figure 7:**
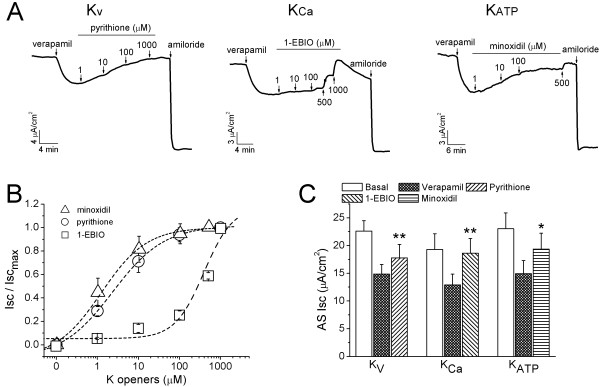
**Stimulatory effects of K^+ ^channel openers subsequent to verapamil in H441 monolayers**. **(A) **Representative traces showing response to a set of K^+ ^channel openers from 1 μM to 1 mM in the presence of verapamil. **(B) **Corresponding concentration-response curves. The raw data were fitted with the Hill equation and the EC_50 _were 2.4, 391.8, and 1.2 μM, respectively, for pyrithione-Na, 1-EBIO, and minoxidil. **(C) **Summary of AS Isc levels before (Basal) and after verapamil (Verapamil) and K^+ ^channel openers (10 μM Pyrithione-Na, 600 μM 1-EBIO, 10 μM Minoxidil). Paired t-test. *P < 0.05, **P < 0.01 for comparison of before and after K^+ ^channel openers. n = 3-6.

Our *in vivo *studies suggest K^+ ^channel openers may alter ENaC-like activity. To address this issue, K^+ ^channel openers were added basolaterally subsequent to verapamil (100 μM) as shown in Fig. 7A. A set of increasing concentrations for pyrithione-Na (K_V7.1 _opener at 5 μM and KCNQ at larger concentrations), 1-EBIO (K _Ca3.1 _opener), and minoxidil (K_ATP _opener) were applied to the basolateral compartment. The average concentration-response curves were plotted in Fig. 7B. The half-maximal effective concentrations (EC_50_) were 2.4 μM, 391.8 μM, and 1.2 μM, respectively, for pyrithione-Na, 1-EBIO, and minoxidil. To maximally activate these K^+ ^channels, the concentration used for each type of K^+ ^channels was based on the results of the dose-response studies (Fig. 7A &7B). As shown in Fig. 7C, pyrithione-Na (10 μM), 1-EBIO (600 μM), and minoxidil (10 μM) significantly increased AS Isc levels from 14.9 ± 1.7 to 17.8 ± 2.4 μA/cm^2 ^(*P *< 0.01, n = 6), 12.9 ± 1.9 to 18.6 ± 2.6 μA/cm^2 ^(*P *< 0.01, n = 6), and 14.9 ± 2.4 to 19.4 ± 2.8 μA/cm^2 ^(*P *< 0.05, n = 3), respectively. These encouraging observations imply that stimulating K^+ ^secretion with K^+ ^channel openers can reverse, at least partially, verapamil-inhibited AS transepithelial Na^+ ^pathways. In fact, this set of experiments was initiated with a low dose of pyrithione-Zn (ZnPy, 10 μM), which was supposed to specifically open heterologously expressed K_V_LQT1 current, one of large K_V _family [50]. Interestingly, only a transient increment was observed followed by a continuing decline in an hour (Additional file 1). This is likely due to the non-specific effects of Zn^2+ ^ions on transepithelial ion transport systems, including ENaC [51-54]. We thus had to utilize its sodium compound, which has a divergent EC_50 _value for native K_V _channels in H441 cells (Fig. 7B).

We also tried to prevent the inhibitory effects of verapamil on the AS Isc levels by addition of K^+ ^channel openers prior to verapamil. The similar transient or sustained elevation in the Isc levels was observed following the application of the K^+ ^channel openers but inexplicably the subsequent application of verapamil inhibited Isc levels to the same extent as that of control monolayers in the absence of K^+ ^channel openers (data not shown). In sharp contrast to the significant recovery effects of verapamil-inhibited ion transport, the K^+ ^channel openers did not prevent the verapamil-induced depression in transepithelial ion transport. These observations indicate that instead of keeping K^+ ^channels from the inhibitory of verapamil, K^+ ^channel openers are only able to recover impaired K^+ ^channel activities.

### Diverse stimulating effects of K^+ ^channel openers on apical and basolateral ion transport

**Figure 8:**
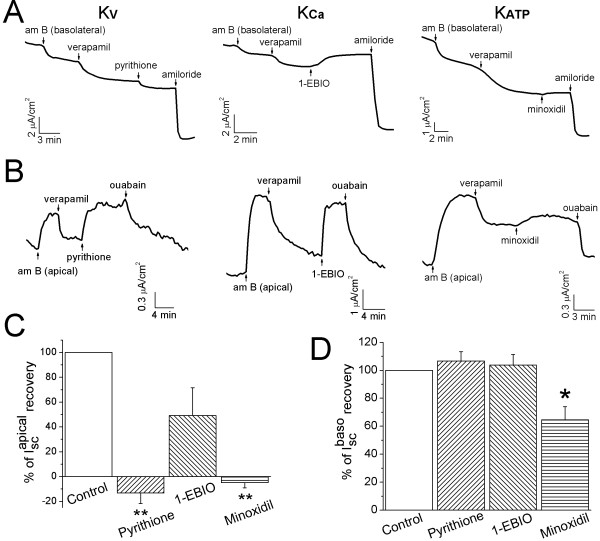
**Compartment-dependent recovery of verapamil-inhibited Isc levels by K^+ ^channel openers in permeabilized H441 monolayers**. **(A) **Representative Isc traces of apical Isc level in the presence of 1 mM ouabain in the basolateral membrane permeabilized monolayers. **(B) **Representative Isc traces of basolateral Isc level in the presence of 100 mM amiloride in the apical permeabilized H441 monolayers. **(C & D) **Recovered apical **(C) **and basolateral **(D) **Isc levels by K^+ ^channel openers. One-way ANOVA for comparing normalized Isc level post K^+ ^channel openers with total AS or OS Isc levels. *P < 0.05 and **P < 0.01. n = 3-5.

Recovery of verapamil-inhibited transepithelial Isc levels in H441 cells (Fig. 7) by the K^+ ^channel openers raised a new question of what Na^+ ^transport systems are regulated by the K^+ ^channel openers, apical ENaC or basolateral Na^+^/K^+^-ATPase. To address this question, K^+ ^channel openers were applied to either apical or basolateral membrane permeabilized monolayers. In basolateral permeabilized cells, neither KCNQ (pyrithione-Na) nor K_ATP _(minoxidil) channel openers altered AS transapical Na^+ ^influx (Fig. 8A &8C). However, K_Ca3.1 _channel opener (1-EBIO) approximately restored half (49.1 ± 22.3%) of the depressed AS Isc level. Regarding transbasolateral ion transport, pyrithione-Na and 1-EBIO but not minoxidil (64.6 ± 9.2%) completely restored the verapamil-inhibited OS Isc levels in apical membrane permeabilized H441 monolayers (Fig. 8B &8D). Taken together, these results suggest that K_Ca3.1 _channels are probably located at both apical and basolateral membranes in H441 cells, while pyrithione- and minoxidil-activated channels may be expressed at the basolateral membrane only.

### Direct regulation of αβγ ENaC by verapamil in *X. laevis *oocytes

**Figure 9:**
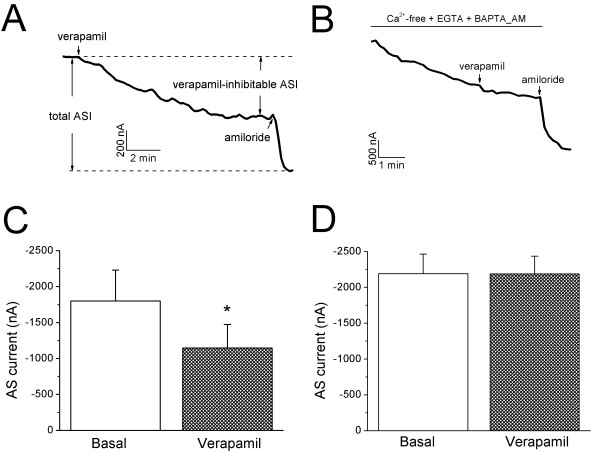
**Verapamil regulates heterologously expressed human αβγ ENaC in oocytes in a Ca^2+^-dependent pattern**. **(A & B) **Time-courses of whole-cell current traces at -100 mV recorded in oocytes perfused with verapamil under normal **(A) **and Ca^2+^-chelated conditions **(B)**. 10 μM amiloride were added at the end of recording to compute basal and verapamil-inhibitable ASI levels. **(C & D) **Effects of verapamil on ENaC activity in control **(C) **and Ca^2+^-chelated **(D) **oocytes. The shift of base line in Ca^2+^-chelated cells **(B) **was corrected for comparing the current levels before (Basal) and after verapamil (Verapamil). Paired t-test. *P < 0.05.

To address the question of whether verapamil directly regulate human ENaC, human α, β, and γ ENaC subunits were co-expressed in *X. laevis *oocytes, and the effects of verapamil on heterologously expressed ENaC were assessed. Verapamil inhibited ENaC current in an oocyte under physiological conditions (Fig. 9A), which is consistent with the results in H441 cells. An average of 38.2 ± 5.5% ENaC currents was reduced by verapamil (*P *< 0.05, n = 4, Fig. 9C), suggesting that verapamil may also directly reduce native ENaC channel activity in H441 cells.

If intracellular Ca^2+ ^signal mediates the down-regulation of αβγ ENaC by verapamil, one may expects that cell permeable Ca^2+ ^chelator could have the same effect. To address this issue, BAPTA_AM was superfused on oocytes bathed in the Ca^2+^-free solution. In an oocyte perfused with the Ca^2+^-free bath solution (5 mM EGTA, 0 mM Ca^2+^), the whole-cell ENaC current declined gradually (Fig. 9B). The residual ENaC currents were no longer sensitive to verapamil under these Ca^2+^-depletion conditions (the ENaC current even increased by 0.4 ± 1.5% after correction of the run-down slope, *P*>0.05 compared with basal current, n = 4, Fig. 9D). Obviously, verapamil down-regulates human αβγ ENaC in a cytosolic Ca^2+^- dependent fashion in oocytes. We also tried to repeat these experiments in Ca^2+ ^depleted H441 monolayers, unfortunately, the resistance and current levels post BAPTA_AM exposure were too low to detect due to impaired gap junctions and ion transport (data not shown).

### Verapamil alters cytosolic Ca^2+ ^signal

**Figure 10:**
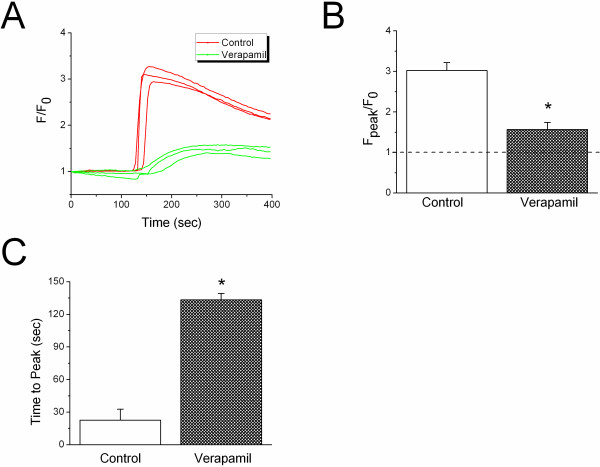
**Verapamil inhibits ionophore-induced Ca^2+ ^mobilization**. Transient Ca^2+ ^signal evoked by ionomycin was measured as relative fluorescent intensity with Fluo 4AM dye in real time with paired manner, in both control and verapamil incubated H441 cells. **(A) **Original traces showing on-time Ca^2+ ^signal digitized in control (red) and verapamil exposed cells (green). **(B) **Maximal relative change in fluorescent intensity (F_peak_/F_0_). Nonparametric Mann-Whitney U-test. *P < 0.05 vs Control. n = 3. **(C) **The time to reach peak signal. Nonparametric Mann-Whitney U-test. *P < 0.05

The regulation of alveolar ENaC by Ca^2+ ^signal has been documented by the well-designed *in vivo *and *in vitro *studies [55,56]. We reason that verapamil may interfere with transepithelial Na^+ ^transport by altering cell Ca^2+ ^signal, which is regulated by mechanic stress associated with breath. The intracellular Ca^2+ ^signal was measured with Fluo 4AM in real time using confocal microscopy (Additional files 1 &2) in H441 cells in the absence or presence of verapamil. Ionomycin was added to the chamber to mimic the Ca^2+ ^wave caused by breath. An approximately three-fold increment in fluorescent intensity was observed following the exposure to ionomycin (Fig. 10A &10B, Additional file 2). In comparison, this increment was significantly diminished in the presence of verapamil (Fig. 10A &10B, Additional file 3). In addition, the time required to reach the maximal value of fluorescent intensity was considerably prolonged in verapamil exposed cells (Fig. 10C, *P *< 0.05).

## Discussion

We aimed to study the cellular mechanistic pathogenesis of the CCB-induced noncardiogenic defect in lung fluid clearance. Ussing chamber studies suggest that transepithelial Na^+ ^transport is inhibited by four structurally distinct subgroups of CCB compounds in human lung epithelial cells (H441 cells). Verapamil reduces amiloride-sensitive (AS) Isc levels in a concentration-dependent manner. Ca^2+ ^signal is involved in the down-regulation of AS Na^+ ^transport by verapamil. Furthermore, verapamil alters K^+ ^recycling via stimulating the apical and basolateral K^+ ^channels as well as Na^+^/K^+^-ATPase activity. K^+ ^channel openers restore the suppressed ENaC activity *in vitro *to a significant extent. Of note, our *in vivo *alveolar fluid clearance (AFC) studies show that K^+ ^channel openers restore the verapamil-inhibited fluid resolution.

A Ca^2+ ^signal has been shown to up-regulate alveolar fluid clearance and epithelial Na^+ ^channel activity [55,57]. Depletion of intracellular Ca^2+ ^by thapsigargin in late-gestational guinea pig lungs completely inhibited amiloride-sensitive AFC [55]. On the other hand, elevation of intraepithelial Ca^2+ ^concentration by β-adrenergic agonists and other AFC-enhancing reagents, for example, terbutaline, has been confirmed [58]. CCB partially blocked terbutaline-stimulated Na^+ ^absorption via amiloride-sensitive channels in primary rat alveolar type II cells [57,59]. Our results that show BAPTA_AM completely abolishes verapamil-induced inhibition of αβγ ENaC activity in oocytes suggest that the regulation of ENaC by CCB is at least partially mediated by an alteration in cytosolic Ca^2+ ^signal (Fig. 10). The observation that verapamil inhibits ionophore-induced Ca^2+ ^mobilization supports this notion. The direct inhibitory effects of Ca^2+ ^ions on ENaC *in vitro *[60,61] were, perhaps, overwhelmed by the stimulatory effects of Ca^2+ ^downstream signals on ENaC and other transporters in these cell models and *in vivo *studies.

**Figure 11:**
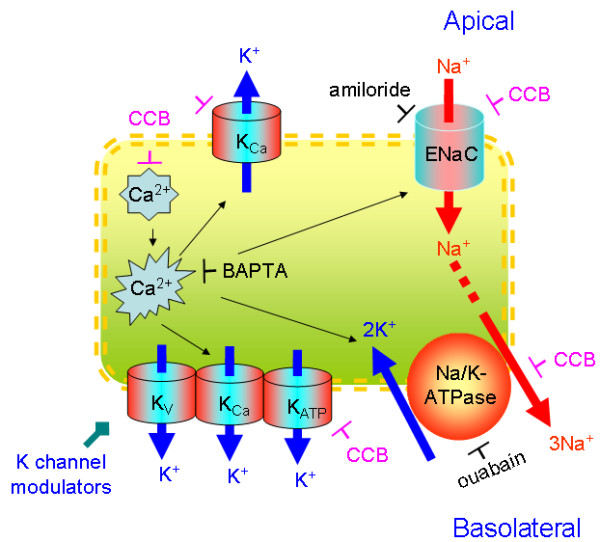
**Schematic model for the multiple mechanisms of CCB-inhibited transepithelial Na^+ ^transport and recovery by K^+ ^channels**. CCB compounds alter Ca^2+ ^signal most likely via modifying Ca^2+ ^release from cytosolic compartments. In addition, CCB compounds directly regulate K^+ ^channels, ENaC, and Na^+^/K^+^-ATPase. Disrupted basolateral K^+ ^recycling and apical ion transport will abrogate transalveolar salt and fluid transport. K^+ ^channel openers significantly restore the CCB-inhibited transepithelial Na^+ ^transport by activating K^+ ^channel, then facilitating Na^+^/K^+^-ATPase.

Accumulating evidence demonstrates that the regulation of epithelial K^+ ^channels by the Ca^2+ ^signal. The expression of various K^+ ^channels has been detected in alveolar and bronchial epithelial cells [20]. Ca^2+ ^signal may regulate those K^+ ^channels by both directly affecting the gating kinetics and serving as a second messenger for signal transduction. On the other hand, the relationship between a Ca^2+ ^signal and Na^+^/K^+^-ATPase is not known. Control of endoplasmic reticulum (ER) Ca^2+ ^release by Na^+^/K^+^-ATPase has been recently confirmed in "knockout" cultured renal epithelial cells [62]. It raises the possibility that CCB may directly inhibit Na^+^/K^+^-ATPase and in turn alter the intracellular Ca^2+ ^content. Nevertheless, our data clearly confirm that impaired K^+ ^ion transport across alveolar basolateral membrane is an essential mechanism for CCB to inhibit ENaC function. Interruption of K^+ ^ion recycling may be a critical mechanism for CCB-induced inhibition of ENaC activity (Fig. 11).

What are the underlying mechanisms for the diverse regulation of apical and basolateral conductance by K^+ ^channel openers? If the K^+ ^channel openers restore the depressed ENaC and Na^+^/K^+^-ATPase by stimulating K^+ ^influx which facilitates Na^+^/K^+^-ATPase in intact cells, no effects on ENaC should be observed in basolateral permeabilized monolayers. Intriguingly, K _Ca3.1 _channel opener still activated ENaC. It is possible that K_Ca _channels are expressed in apical membrane [29]. Increased extrusion of K^+ ^ions in the presence of 1-EBIO may locally build up an electrical gradient resulting in elevated ENaC activity. Another possibility is that 1-EBIO directly stimulates ENaC. The less effect of minoxidil in permeabilized monolayers, by comparison to the results in intact H441 cells, *in vivo *fluid clearance, and previous publication [29], may be due to loss of cell ATP.

CCB compounds may not act on the same Ca^2+ ^entry/exit pathways due to their divergent pharmaceutical properties [63]. Lung epithelial Ca^2+ ^content is determined by Ca^2+ ^influx/efflux pathways, endoplasmic reticulum (ER) Ca^2+ ^release, and concentration of Ca^2+^-binding proteins. Ca^2+ ^ions may enter epithelia through, for example, L-type Ca^2+ ^channels, ECaC, and other TRP channels; while Ca^2+^-ATPase and Ca^2+^/Na^+ ^exchanger are major transporters to extrude Ca^2+ ^ions. Our results indicate that verapamil may not alter basal Ca^2+ ^content, instead, the Ca^2+ ^wave, possibly due to Ca^2+ ^release from cytosolic compartments, was abolished (Fig. 11).

The potent efficacy of K^+ ^channel openers to recover transepithelial Na^+ ^reabsorption and fluid clearance may be a promising therapeutic approach to mitigate drug-induced as well as other deleterious agents-induced noncardiogenic lung edema. On the other hand, inhalation CCB compounds will definitely bring life-threatening noncardiogenic lung edema to patients. Reasonably, any medicines capable of depleting lung epithelial Ca^2+ ^ions may have the same fatal side-effect when delivered either intravenously or intratracheally. Indeed, nifedipine cannot prevent lung edema in mountain sickness [64].

As supported by our observations in permeabilized H441 monolayers and Ca^2+^-depleted cells, verapamil inhibited basolateral and apical K^+ ^conductance directly. Verapamil inhibits IK (K_Ca3.1_) channels with an IC_50 _value of 72 μM, various K_V _channel subtypes between 10-200 μM and K_ATP _channels at ~10 μM [65-67]. Moreover, concentrations typically used to achieve nearly complete inhibition of voltage-gated Ca^2+ ^channels is less than 100 μM. Therefore, it is most likely that verapamil alters ENaC activity via multiple mechanisms, for example, through Ca^2+^-mediated regulation, direct inhibition of ENaC, and interrupting K^+ ^recycling (Fig. 11). In summary, CCB reagents decrease vectorial transepithelial Na^+ ^transport directly by inhibiting apical ENaC and indirectly by altering cytosolic Ca^2+ ^signal and K^+ ^recycling at the basolateral membrane. Recovery of the CCB-depressed edema resolution by K^+ ^channel openers indicates that pharmaceutical augmentation of K^+ ^recycling may be a potent strategy to combat CCB-induced noncardiogenic lung edema.

## Competing interests

The authors declare that they have no competing interests.

## Authors' contributions

DYH and HGN performed Ussing chamber and voltage clamp studies and analyzed data. XG carried out *in vivo *alveolar fluid clearance. DYH and RCN detected the intracellular Ca^2+ ^intensity. XFS and YC prepared cRNA and voltage clamp recording. HLJ, JF, and VR designed experiments, analyzed data, and prepared manuscript. All authors have read and approved the final manuscript.

## Supplementary Material

Additional file 1 **Pyrithione zinc on K_V _in H441 cells**. The specific blocker for heterologously expressed K_V _channels, pyrithione zinc transiently actives AS Isc followed by a pronounced decline. The remaining Isc level is approximately 0 for AS Isc fraction. Click here for file

Additional file 2 **Video clip in a control H441 cell**. Fluo 4AM fluorescent intensity was monitored in real time before and after addition of ionomycin. Click here for file

Additional file 3 **Video clip in a verapamil incubated H441 cell**. Fluo 4AM fluorescent intensity was monitored in real time before and after addition of ionomycin. Click here for file

## References

[B1] Sami Karti S Ulusoy H Yandi M Gunduz A Kosucu M Erol K Ratip S Non-cardiogenic pulmonary oedema in the course of verapamil intoxication Emerg Med J 2002 19 5 458 459 10.1136/emj.19.5.458 12205007 PMC1725949

[B2] Nassar AH Ghazeeri G Usta IM Nifedipine-associated pulmonary complications in pregnancy Int J Gynaecol Obstet 2007 97 2 148 149 10.1016/j.ijgo.2007.01.011 17368643

[B3] Vaast P Dubreucq-Fossaert S Houfflin-Debarge V Provost-Helou N Ducloy-Bouthors AS Puech F Subtil D Acute pulmonary oedema during nicardipine therapy for premature labour; Report of five cases Eur J Obstet Gynecol Reprod Biol 2004 113 1 98 99 10.1016/j.ejogrb.2003.05.004 15036720

[B4] Abbas OM Nassar AH Kanj NA Usta IM Acute pulmonary edema during tocolytic therapy with nifedipine Am J Obstet Gynecol 2006 195 4 e3 4 10.1016/j.ajog.2006.06.032 16846586

[B5] Janower S Carbonne B Lejeune V Apfelbaum D Boccara F Cohen A Acute pulmonary edema during preterm labor: role of nicardipine tocolysis (three cases) J Gynecol Obstet Biol Reprod (Paris) 2005 34 8 807 812 16319773 10.1016/s0368-2315(05)82958-8

[B6] Chapuis C Menthonnex E Debaty G Koch FX Rancurel E Menthonnex P Pons JC Acute pulmonary edema during nicardipine and salbutamol therapy for preterm labor in twin pregnancy J Gynecol Obstet Biol Reprod (Paris) 2005 34 5 493 496 16142141 10.1016/s0368-2315(05)82858-3

[B7] Stanek EJ Nelson CE DeNofrio D Amlodipine overdose Ann Pharmacother 1997 31 7-8 853 856 9220044 10.1177/106002809703100708

[B8] Humbert VH Jr Munn NJ Hawkins RF Noncardiogenic pulmonary edema complicating massive diltiazem overdose Chest 1991 99 1 258 259 10.1378/chest.99.1.258 1984972

[B9] Brass BJ Winchester-Penny S Lipper BL Massive verapamil overdose complicated by noncardiogenic pulmonary edema Am J Emerg Med 1996 14 5 459 461 10.1016/S0735-6757(96)90151-5 8765109

[B10] Matthay MA Folkesson HG Clerici C Lung epithelial fluid transport and the resolution of pulmonary edema Physiol Rev 2002 82 569 600 12087129 10.1152/physrev.00003.2002

[B11] Eaton DC Helms MN Koval M Bao HF Jain L The Contribution of Epithelial Sodium Channels to Alveolar Function in Health and Disease Annu Rev Physiol 2009 71 403 423 10.1146/annurev.physiol.010908.163250 18831683

[B12] Matalon S Lazrak A Jain L Eaton DC Invited review: biophysical properties of sodium channels in lung alveolar epithelial cells J Appl Physiol 2002 93 5 1852 1859 12381774 10.1152/japplphysiol.01241.2001

[B13] Zemans RL Matthay MA Bench-to-bedside review: the role of the alveolar epithelium in the resolution of pulmonary edema in acute lung injury Crit Care 2004 8 6 469 477 10.1186/cc2906 15566618 PMC1065044

[B14] Matthay MA Robriquet L Fang X Alveolar epithelium: role in lung fluid balance and acute lung injury Proc Am Thorac Soc 2005 2 3 206 213 10.1513/pats.200501-009AC 16222039

[B15] Hummler E Barker P Gatzy J Beermann F Verdumo C Schmidt A Boucher R Rossier BC Early death due to defective neonatal lung liquid clearance in α-ENaC-deficient mice Nat Genet 1996 12 3 325 328 10.1038/ng0396-325 8589728

[B16] Chen L Song W Davis IC Shrestha K Schwiebert E Sullender WM Matalon S Inhibition of Na^+ ^transport in lung epithelial cells by respiratory syncytial virus infection Am J Respir Cell Mol Biol 2009 40 5 588 600 10.1165/rcmb.2008-0034OC 18952569 PMC2677438

[B17] Rossier BC Pradervand S Schild L Hummler E Epithelial sodium channel and the control of sodium balance: interaction between genetic and environmental factors Annu Rev Physiol 2002 64 877 897 10.1146/annurev.physiol.64.082101.143243 11826291

[B18] O'Grady SM Lee SY Molecular diversity and function of voltage-gated (Kv) potassium channels in epithelial cells Int J Biochem Cell Biol 2005 37 8 1578 1594 10.1016/j.biocel.2005.04.002 15882958

[B19] Inglis SK Brown SG Constable MJ McTavish N Olver RE Wilson SM A Ba^2+^-resistant, acid-sensitive K^+ ^conductance in Na^+^-absorbing H441 human airway epithelial cells Am J Physiol Lung Cell Mol Physiol 2007 292 5 L1304 1312 10.1152/ajplung.00424.2006 17277046 PMC2136209

[B20] O'Grady SM Lee SY Chloride and potassium channel function in alveolar epithelial cells Am J Physiol Lung Cell Mol Physiol 2003 284 5 L689 700 12676759 10.1152/ajplung.00256.2002

[B21] Leroy C Dagenais A Berthiaume Y Brochiero E Molecular identity and function in transepithelial transport of K(ATP) channels in alveolar epithelial cells Am J Physiol Lung Cell Mol Physiol 2004 286 5 L1027 1037 10.1152/ajplung.00249.2003 14729507

[B22] Bardou O Trinh NT Brochiero E Molecular diversity and function of K^+ ^channels in airway and alveolar epithelial cells Am J Physiol Lung Cell Mol Physiol 2008 19060226 10.1152/ajplung.90525.2008

[B23] Greenwood IA Yeung SY Hettiarachi S Andersson M Baines DL KCNQ-encoded channels regulate Na^+ ^transport across H441 lung epithelial cells Pflugers Arch 2009 457 4 785 794 10.1007/s00424-008-0557-7 18663467

[B24] Bernard K Bogliolo S Soriani O Ehrenfeld J Modulation of calcium-dependent chloride secretion by basolateral SK4-like channels in a human bronchial cell line J Membr Biol 2003 196 1 15 31 10.1007/s00232-003-0621-3 14724753

[B25] Leroy C Prive A Bourret JC Berthiaume Y Ferraro P Brochiero E Regulation of ENaC and CFTR expression with K^+ ^channel modulators and effect on fluid absorption across alveolar epithelial cells Am J Physiol Lung Cell Mol Physiol 2006 291 6 L1207 1219 10.1152/ajplung.00376.2005 16891388

[B26] Szkotak AJ Ng AM Sawicka J Baldwin SA Man SF Cass CE Young JD Duszyk M Regulation of K^+ ^current in human airway epithelial cells by exogenous and autocrine adenosine Am J Physiol Cell Physiol 2001 281 6 C1991 2002 11698258 10.1152/ajpcell.2001.281.6.C1991

[B27] Monaghan AS Baines DL Kemp PJ Olver RE Inwardly rectifying K^+ ^currents of alveolar type II cells isolated from fetal guinea-pig lung: regulation by G protein- and Mg^2+^-dependent pathways Pflugers Arch 1997 433 3 294 303 10.1007/s004240050280 9064645

[B28] Berthiaume Y Folkesson HG Matthay MA Lung edema clearance: 20 years of progress: invited review: alveolar edema fluid clearance in the injured lung J Appl Physiol 2002 93 6 2207 2213 12433940 10.1152/japplphysiol.01201.2001

[B29] Sakuma T Takahashi K Ohya N Nakada T Matthay MA Effects of ATP-sensitive potassium channel opener on potassium transport and alveolar fluid clearance in the resected human lung Pharmacol Toxicol 1998 83 1 16 22 10.1111/j.1600-0773.1998.tb01436.x 9764421

[B30] Peth S Karle C Dehnert C Bartsch P Mairbaurl H K^+ ^channel activation with minoxidil stimulates nasal-epithelial ion transport and blunts exaggerated hypoxic pulmonary hypertension High Alt Med Biol 2006 7 1 54 63 10.1089/ham.2006.7.54 16544967

[B31] Briot R Frank JA Uchida T Lee JW Calfee CS Matthay MA Elevated levels of the receptor for advanced glycation end products, a marker of alveolar epithelial type I cell injury, predict impaired alveolar fluid clearance in isolated perfused human lungs Chest 2009 135 2 269 275 10.1378/chest.08-0919 19017890 PMC2714162

[B32] Factor P Mutlu GM Chen L Mohameed J Akhmedov AT Meng FJ Jilling T Lewis ER Johnson MD Xu A Adenosine regulation of alveolar fluid clearance Proc Natl Acad Sci USA 2007 104 10 4083 4088 10.1073/pnas.0601117104 17360481 PMC1820712

[B33] Lazrak A Nita I Subramaniyam D Wei S Song W Ji HL Janciauskiene S Matalon S α1-antitrypsin inhibits epithelial Na^+ ^transport *in vitro *and *in vivo* Am J Respir Cell Mol Biol 2009 41 3 261 270 10.1165/rcmb.2008-0384OC 19131639 PMC2742747

[B34] Gu X Wang Z Xu J Maeda S Sugita M Sagawa M Toga H Sakuma T Denopamine stimulates alveolar fluid clearance via cystic fibrosis transmembrane conductance regulator in rat lungs Respirology 2006 11 5 566 571 10.1111/j.1440-1843.2006.00898.x 16916328

[B35] Chen L Patel RP Teng X Bosworth CA Lancaster JR Jr Matalon S Mechanisms of cystic fibrosis transmembrane conductance regulator activation by S-nitrosoglutathione J Biol Chem 2006 281 14 9190 9199 10.1074/jbc.M513231200 16421103

[B36] Thome U Chen L Factor P Dumasius V Freeman B Sznajder JI Matalon S Na, K-ATPase gene transfer mitigates an oxidant-induced decrease of active sodium transport in rat fetal ATII cells Am J Respir Cell Mol Biol 2001 24 3 245 252 11245623 10.1165/ajrcmb.24.3.3994

[B37] Thome UH Davis IC Nguyen SV Shelton BJ Matalon S Modulation of sodium transport in fetal alveolar epithelial cells by oxygen and corticosterone Am J Physiol Lung Cell Mol Physiol 2003 284 2 L376 385 12533313 10.1152/ajplung.00218.2002

[B38] Guo Y DuVall MD Crow JP Matalon S Nitric oxide inhibits Na^+ ^absorption across cultured alveolar type II monolayers Am J Physiol 1998 274 3 Pt 1 L369 377 9530172 10.1152/ajplung.1998.274.3.L369

[B39] Kirk KL Halm DR Dawson DC Active sodium transport by turtle colon via an electrogenic Na-K exchange pump Nature 1980 287 5779 237 239 10.1038/287237a0 6253798

[B40] Ji HL Su XF Kedar S Li J Barbry P Smith PR Matalon S Benos DJ δ-subunit confers novel biophysical features to αβγ-human epithelial sodium channel (ENaC) via a physical interaction J Biol Chem 2006 281 12 8233 8241 10.1074/jbc.M512293200 16423824

[B41] Musa-Aziz R Oliveira-Souza M Mello-Aires M Signaling pathways in the biphasic effect of ANG II on Na^+^/H^+ ^exchanger in T84 cells J Membr Biol 2005 205 2 49 60 10.1007/s00232-005-0762-7 16283585

[B42] Espelt MV Estevez AY Yin X Strange K Oscillatory Ca^2+ ^signaling in the isolated *Caenorhabditis elegans *intestine: role of the inositol-1,4,5-trisphosphate receptor and phospholipases C β and γ J Gen Physiol 2005 126 4 379 392 10.1085/jgp.200509355 16186564 PMC2266627

[B43] Pendurthi UR Ngyuen M Andrade-Gordon P Petersen LC Rao LV Plasmin induces Cyr61 gene expression in fibroblasts via protease-activated receptor-1 and p44/42 mitogen-activated protein kinase-dependent signaling pathway Arterioscler Thromb Vasc Biol 2002 22 9 1421 1426 10.1161/01.ATV.0000030200.59331.3F 12231560

[B44] Praetorius HA Leipziger J Released nucleotides amplify the cilium-dependent, flow-induced [Ca^2+^]_i _response in MDCK cells Acta Physiol (Oxf) 2009 197 3 241 251 10.1111/j.1748-1716.2009.02002.x 19432587

[B45] Lazrak A Matalon S cAMP-induced changes of apical membrane potentials of confluent H441 monolayers Am J Physiol Lung Cell Mol Physiol 2003 285 2 L443 450 12704021 10.1152/ajplung.00412.2002

[B46] Thomas CP Campbell JR Wright PJ Husted RF cAMP-stimulated Na^+ ^transport in H441 distal lung epithelial cells: role of PKA, phosphatidylinositol 3-kinase, and sgk1 Am J Physiol Lung Cell Mol Physiol 2004 287 4 L843 851 10.1152/ajplung.00340.2003 15208094

[B47] Nie HG Chen L Han DY Li J Song WF Wei SP Fang XH Gu X Matalon S Ji HL Regulation of epithelial sodium channels by cGMP/PKGII J Physiol 2009 587 Pt 11 2663 2676 10.1113/jphysiol.2009.170324 19359370 PMC2714029

[B48] Woollhead AM Baines DL Forskolin-induced cell shrinkage and apical translocation of functional enhanced green fluorescent protein-human αENaC in H441 lung epithelial cell monolayers J Biol Chem 2006 281 8 5158 5168 10.1074/jbc.M509947200 16373340

[B49] Zhang S Zhou Z Gong Q Makielski JC January CT Mechanism of block and identification of the verapamil binding domain to HERG potassium channels Circ Res 1999 84 9 989 998 10325236 10.1161/01.res.84.9.989

[B50] Gao Y Chotoo CK Balut CM Sun F Bailey MA Devor DC Role of S3 and S4 transmembrane domain charged amino acids in channel biogenesis and gating of KCa2.3 and KCa3.1 J Biol Chem 2008 283 14 9049 9059 10.1074/jbc.M708022200 18227067 PMC2431042

[B51] Bancila V Cens T Monnier D Chanson F Faure C Dunant Y Bloc A Two SUR1-specific histidine residues mandatory for zinc-induced activation of the rat KATP channel J Biol Chem 2005 280 10 8793 8799 10.1074/jbc.M413426200 15613469

[B52] Swaminath G Lee TW Kobilka B Identification of an allosteric binding site for Zn^2+ ^on the β2 adrenergic receptor J Biol Chem 2003 278 1 352 356 10.1074/jbc.M206424200 12409304

[B53] Amuzescu B Segal A Flonta ML Simaels J Van Driessche W Zinc is a voltage-dependent blocker of native and heterologously expressed epithelial Na^+ ^channels Pflugers Arch 2003 446 1 69 77 12690465 10.1007/s00424-002-0998-3

[B54] Bossy-Wetzel E Talantova MV Lee WD Scholzke MN Harrop A Mathews E Gotz T Han J Ellisman MH Perkins GA Crosstalk between nitric oxide and zinc pathways to neuronal cell death involving mitochondrial dysfunction and p38-activated K^+ ^channels Neuron 2004 41 3 351 365 10.1016/S0896-6273(04)00015-7 14766175

[B55] Norlin A Folkesson HG Ca^2+^-dependent stimulation of alveolar fluid clearance in near-term fetal guinea pigs Am J Physiol Lung Cell Mol Physiol 2002 282 4 L642 649 11880288 10.1152/ajplung.00417.2000

[B56] Swystun V Chen L Factor P Siroky B Bell PD Matalon S Apical trypsin increases ion transport and resistance by a phospholipase C-dependent rise of Ca^2+^ Am J Physiol Lung Cell Mol Physiol 2005 288 5 L820 830 10.1152/ajplung.00396.2004 15626748

[B57] Marunaka Y Niisato N Effects of Ca^2+ ^channel blockers on amiloride-sensitive Na^+ ^permeable channels and Na^+ ^transport in fetal rat alveolar type II epithelium Biochem Pharmacol 2002 63 8 1547 1552 10.1016/S0006-2952(02)00880-8 11996897

[B58] Niisato N Nakahari T Tanswell AK Marunaka Y β2-agonist regulation of cell volume in fetal distal lung epithelium by cAMP-independent Ca^2+ ^release from intracellular stores Can J Physiol Pharmacol 1997 75 8 1030 1033 10.1139/cjpp-75-8-1030 9360019

[B59] Marunaka Y Niisato N O'Brodovich H Eaton DC Regulation of an amiloride-sensitive Na^+^-permeable channel by a β2-adrenergic agonist, cytosolic Ca^2+ ^and Cl^- ^in fetal rat alveolar epithelium J Physiol 1999 515 Pt 3 669 683 10.1111/j.1469-7793.1999.669ab.x 10066896 PMC2269183

[B60] Ismailov II Berdiev BK Shlyonsky VG Benos DJ Mechanosensitivity of an epithelial Na^+ ^channel in planar lipid bilayers: release from Ca^2+ ^block Biophys J 1997 72 3 1182 1192 10.1016/S0006-3495(97)78766-6 9138565 PMC1184502

[B61] Schild L Schneeberger E Gautschi I Firsov D Identification of amino acid residues in the α, β, and γ subunits of the epithelial sodium channel (ENaC) involved in amiloride block and ion permeation J Gen Physiol 1997 109 1 15 26 10.1085/jgp.109.1.15 8997662 PMC2217053

[B62] Chen Y Cai T Yang C Turner DA Giovannucci DR Xie Z Regulation of inositol 1,4,5-trisphosphate receptor-mediated calcium release by the Na/K-ATPase in cultured renal epithelial cells J Biol Chem 2008 283 2 1128 1136 10.1074/jbc.M708025200 17993456

[B63] DeWitt CR Waksman JC Pharmacology, pathophysiology and management of calcium channel blocker and β-blocker toxicity Toxicol Rev 2004 23 4 223 238 10.2165/00139709-200423040-00003 15898828

[B64] Hohenhaus E Niroomand F Goerre S Vock P Oelz O Bartsch P Nifedipine does not prevent acute mountain sickness Am J Respir Crit Care Med 1994 150 3 857 860 8087361 10.1164/ajrccm.150.3.8087361

[B65] Ninomiya T Takano M Haruna T Kono Y Horie M Verapamil, a Ca^2+ ^entry blocker, targets the pore-forming subunit of cardiac type KATP channel (Kir6.2) J Cardiovasc Pharmacol 2003 42 2 161 168 10.1097/00005344-200308000-00002 12883317

[B66] Pancrazio JJ Viglione MP Kleiman RJ Kim YI Verapamil-induced blockade of voltage-activated K^+ ^current in small-cell lung cancer cells J Pharmacol Exp Ther 1991 257 1 184 191 1850464

[B67] Waldegger S Niemeyer G Morike K Wagner CA Suessbrich H Busch AE Lang F Eichelbaum M Effect of verapamil enantiomers and metabolites on cardiac K^+ ^channels expressed in *Xenopus *oocytes Cell Physiol Biochem 1999 9 2 81 89 10.1159/000016304 10394001

