# Supplementary figures and images for: K+ channel openers restore verapamil-inhibited lung fluid resolution and transepithelial ion transport
Source: Respir Res. 2010 May 27;11(1):65. doi: 10.1186/1465-9921-11-65 (PMC2889873; doi:10.1186/1465-9921-11-65)

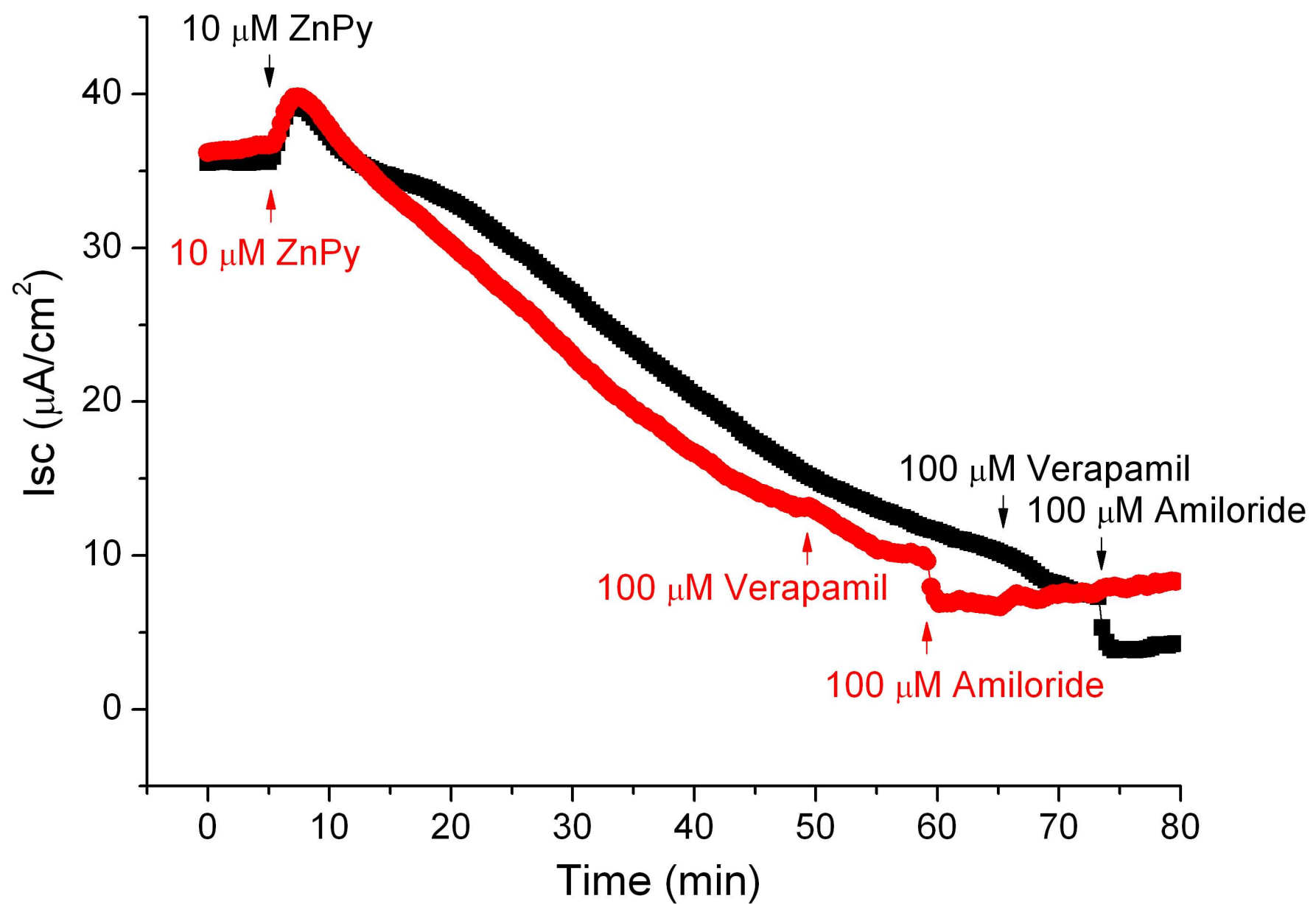

Supplement: Additional file 3 — Video clip in a verapamil incubated H441 cell. Fluo 4AM fluorescent intensity was monitored in real time before and after addition of ionomycin. [file 1465-9921-11-65-S3.PDF]
